# Inflammation Downregulates UCP1 Expression in Brown Adipocytes Potentially via SIRT1 and DBC1 Interaction

**DOI:** 10.3390/ijms18051006

**Published:** 2017-05-08

**Authors:** Mark K. Nøhr, Natalia Bobba, Bjørn Richelsen, Sten Lund, Steen B. Pedersen

**Affiliations:** 1 Institute of Clinical Medicine, Aarhus University, 8000 Aarhus C, Denmark; bjoern.richelsen@aarhus.rm.dk (B.R.); sl@dadlnet.dk (S.L.); steen.pedersen@clin.au.dk (S.B.P.); 2 Department of Endocrinology and Internal Medicine, Aarhus University Hospital, 8000 Aarhus C, Denmark; 3 Laboratory of Metabolic Diseases and Aging, Institut Pasteur Montevideo, Mataojo 2020, 11400 Montevideo, Uruguay; mnbobba@gmail.com

**Keywords:** IL1β, LPS, BAT, UCP1, DBC1, obesity, SIRT1

## Abstract

Brown adipose tissue thermogenesis at the cost of energy is not only important for the development of obesity, but also possesses great promise in anti-obesity treatment. Uncoupling protein 1 (UCP1) expression has been reported to be under control of the intracellular deacetylase SIRT1. Here, we investigated the effect and mechanism of inflammation and sirtuin-1 (SIRT1) activation on the induction of thermogenic genes in immortalized brown adipocytes incubated with LPS or IL1β and mice with elevated inflammatory tone. In vitro stimulation of brown adipocytes with dibutyryl cyclic adenosine monophosthate (dbcAMP) reduced the expression of *deleted in breast cancer-1* (*Dbc1*) (SIRT1 inhibitor) and increased the *Ucp1* expression. Silencing of SIRT1 attenuated dbcAMP induction of *Ucp1*. In contrast, IL1β increased the expression of *Dbc1* and greatly reduced the induction of *Ucp1*. Similarly, in vivo studies revealed decreased expression of *Ucp1* in brown adipose tissue (BAT) in mice chronically infused with LPS. Resveratrol, a known SIRT1 activator, partly rescued the *Ucp1* downregulation by inflammation in both the cell cultures and mice. Here, we describe how the expression of *Ucp1* in BAT is controlled via SIRT1 and is reduced under inflammation and can be rescued by SIRT1 activation by resveratrol. We suggest the reduced UCP1 expression under inflammation is mediated by the increased expression of DBC1, which inhibits SIRT1 activity.

## 1. Introduction

Obesity is, today, a major health concern affecting millions of people worldwide. With obesity, a number of disorders, such as low-grade inflammation, insulin resistance, and type 2 diabetes, are seen [1].

Obesity-associated low-grade inflammation is a potential contributor of insulin resistance [2,3,4]. However, the eliciting factor of low-grade inflammation is not currently known, though adipose tissue hypoxia [5], free fatty acids [6,7], and metabolic endotoxemia [8] have been mentioned. Metabolic endotoxemia is presumably caused by “leaky” gut epithelium causing lipopolysaccharides (LPSs) originating from Gram-negative gut bacteria to enter the systemic circulation. LPS binds to Toll-like receptor 4 (TLR4) on innate immune cells and signals nuclear factor κB (NF-κB) translocation to the nucleus and transcription of proinflammatory cytokines [9]. White adipocytes express TLR4 [6] and are, as such, important immunomodulators. In recent reports, immortalized murine brown adipocytes were reported to express TLR4 and cytokine production when stimulated with LPS [10,11].

In the hunt for new effective anti-obesity treatments, brown adipose tissue (BAT) manipulation has become an attractive candidate. Brown adipocytes express the mitochondrial protein uncoupling protein 1 (UCP1) which uncouples the respiratory chain and thereby generates heat (non-shivering thermogenesis). Thus, instead of storing energy, as seen in white adipose tissue (WAT), BAT burns energy in a process which could be exploited therapeutically. Originally, BAT was believed to be present only in newborns, and gradually lost thereafter. However, later evidence identified areas in, e.g., the neck region of adult humans which were reported as BAT [12,13,14]. Despite the rather limited amount of BAT in adult humans, WAT has a high degree of plasticity and can undergo a “browning” process, i.e., increased expression of BAT-related genes, such as *Ucp1* [15].

Opposite to increasing the amount of BAT and UCP1 expression, having none, or reduced, BAT could also be a contributing factor in the development of obesity. Indeed, the probability of having BAT is inversely correlated with age and body weight [12]. Thus, efforts are being made to elucidate potential mechanisms by which the BAT activity is reduced. In the context of obesity, proinflammatory cytokines, such as interleukin-1β (IL1β) and tumor necrosis factor α (TNFα), or endotoxins (LPS) from the intestine, could influence the expression of thermogenic genes in BAT cells. Furthermore, it was recently shown that reduced browning of white adipose tissue is directly regulated by macrophages docking on adipocytes via integrin α_4_ and its receptor vascular cell adhesion protein 1 [16]. Additionally, UCP1 was recently shown to be downregulated in brown adipocytes treated with LPS [10].

In WAT, the induction of BAT-associated genes (*Ucp1*, *Cidea*, *Dio2*) is under the control of sirtuin-1 (SIRT1)-dependent deacetylation of the transcription factor peroxisome proliferator-activated receptor-γ (PPARγ) [17]. SIRT1 is a NAD^+^-dependent intracellular deacetylase with pleiotropic effects, such as inhibition of NF-κB activity [18] and stimulation of PPARγ co-activator 1α (PGC1α) activity [19] in addition to the already-mentioned PPARγ deacetylation [17]. Additionally, the deletion of the endogenous intracellular SIRT1 inhibitor deleted in breast cancer-1 (DBC1) [20,21] resulted in significant upregulation of brown genes in WAT [17].

Despite the mechanism not being fully established [22,23,24,25], the naturally-occurring (found especially in red wine) polyphenol resveratrol enhances the activity of SIRT1. This results in increased UCP1 expression in HIB-1B brown adipocytes [17].

In the present study, we investigated the expression of brown genes in BAT harvested from a murine model of low-grade inflammation in which LPS was continuously infused via osmotic mini-pumps for 28 days. Furthermore, utilizing immortalized brown adipocytes, we studied regulatory mechanisms of brown genes during inflammation induced by IL1β and low-dose LPS. In addition, resveratrol was tested for its effects on brown adipocyte activity.

## 2. Results

### 2.1. Inflammation Reduces Expression of Ucp1 in Mature Brown Adipocytes

**Figure 1:**
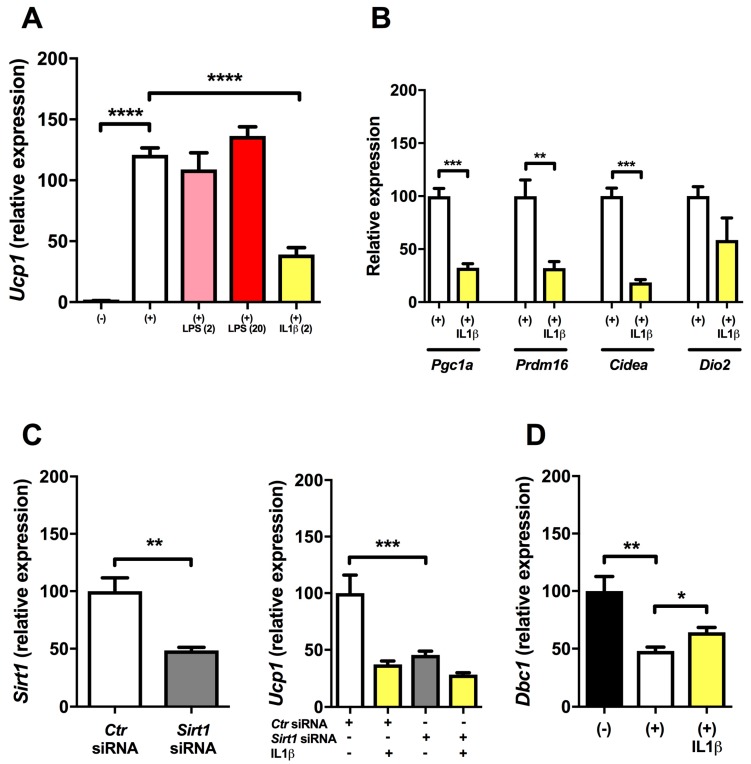
Effects of lipopolysaccharide (LPS) and interleukin 1β (IL1β) on the induction of brown genes in cultured immortalized brown adipocytes. (**A**) 4 h stimulation with dibutyryl cyclic adenosine monophosthate (dbcAMP) significantly induced *Ucp1* expression in mature brown adipocytes. Incubation with low-doses (2 and 20 ng/mL) of LPS did not affect the induction of *Ucp1*. Incubation with IL1β (2 ng/mL) significantly reduced the induction of *Ucp1* (*n* = 6); (**B**) IL1β incubation also affected the induction other brown adipose tissue (BAT)-associated genes such as *Pgc1a*, *Prdm16*, *Cidea*. *Dio2* showed a non-significant trend towards reduced expression by IL1β (*n* = 6); (**C**) SIRT1 silencing by *Sirt1* siRNA significantly reduced *Ucp1* induction (*n* = 6); (**D**) *Dbc1* was reduced in stimulated brown adipocytes and significantly (Student’s *t*-test) upregulated with additional IL1β incubation. * denotes *p* < 0.05, ** denotes *p* < 0.01, *** denotes *p* < 0.001, **** denotes *p* < 0.0001 according to one-way ANOVA followed by Bonferroni’s post hoc analysis or Student’s *t*-test.

To investigate inflammatory effects on *Ucp1* expression, we used immortalized brown adipocytes to test their ability to induce brown genes after incubation with low-dose LPS or IL1β. As *Ucp1* expression is induced by cAMP in BAT, we stimulated cells 4 h with dibutyryl cyclic adenosine monophosthate (dbcAMP) to induce *Ucp1* [26]. Following stimulation with dbcAMP, *Ucp1* expression increased dramatically (Figure 1A). To mimic metabolic low-grade inflammation, we tested rather low concentrations of LPS. However, neither 2 nor 20 ng/mL of LPS affected the expression of *Ucp1* (Figure 1A). Oppositely, IL1β at a concentration of 2 ng/mL greatly reduced the induction of *Ucp1* (Figure 1A). In addition, IL1β reduced the induction of the brown genes *Prdm16* and *Cidea* together with *Pgc1a* important for mitochondrial biogenesis (Figure 1B). Additionally, there was a non-significant trend towards reduced *Dio2* expression by IL1β (Figure 1B).

### 2.2. Regulation of Ucp1 Expression (dbcAMP and SIRT1 Knock-down Experiments)

In our experiments dbcAMP increased *Ucp1* expression ≈ 120-fold (*p* < 0.0001). Previously, SIRT1 activity has been described to be an important regulator of brown remodeling in WAT, including increased expression of UCP1 [17]. In support of this view, we found that partial knock-down of *Sirt1* expression by *Sirt1* siRNA in mature brown adipocytes resulted in reduced induction of *Ucp1* (Figure 1C) by dbcAMP. As SIRT1 activity is regulated at the molecular enzymatic level rather than expressional level [27] by DBC1 [21], we investigated the expression of the endogenous SIRT1 inhibitor DBC1 in our system. When cells were stimulated with dbcAMP, there was a 50% reduction in *Dbc1* expression, which was partly reversed by IL1β (Student’s *t*-test; Figure 1D) resembling the reciprocal expression of *Ucp1* (Figure 1A).

### 2.3. Resveratrol Partly Rescues Ucp1 Downregulation by IL1β

**Figure 2:**
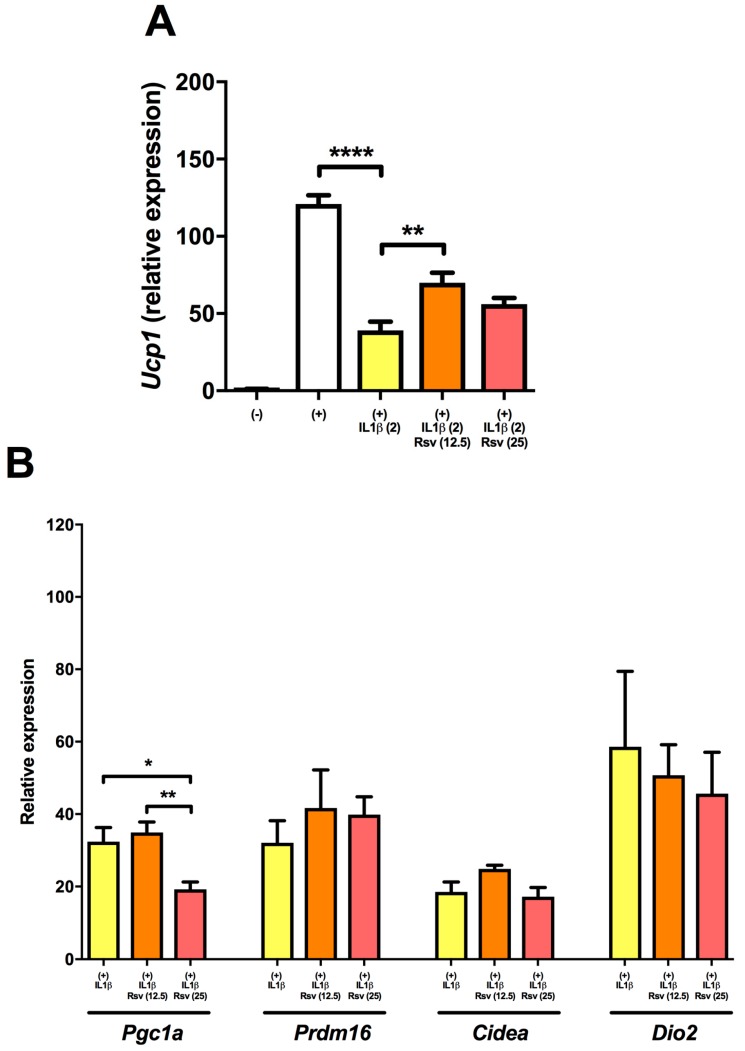
Resveratrol partly rescues IL1β-induced downregulation of *Ucp1*. (**A**) Resveratrol at 12.5 and 25 μM reduced IL1β-mediated downregulation of *Ucp1* (*n* = 5 to 6); (**B**) Resveratrol showed no ameliorating effects on IL1β-mediated downregulation of *Pgc1a*, *Prdm16*, *Cidea*, and *Dio2* (*n* = 5 to 6). Resveratrol at 25 μM further downregulated the expression of *Pgc1a*. * denotes *p* < 0.05 ** denotes *p* < 0.01, **** denotes *p* < 0.0001 according to one-way ANOVA followed by Bonferroni’s post hoc analysis.

Though the mechanism is not precisely known, resveratrol has been described to enhance the activity of SIRT1. We, therefore, wanted to investigate whether resveratrol could rescue the IL1β-induced decline in *Ucp1* expression. Both 12.5 and 25 μM of resveratrol partly reduced the downregulation of *Ucp1* induced by IL1β (Figure 2A). The former concentration showed the highest effect. Resveratrol showed no rescuing effect of *Pgc1a* expression or the brown genes *Prdm16*, *Cidea*, and *Dio2* and 25 μM resveratrol actually further downregulated *Pgc1a* (Figure 2B).

### 2.4. Effects of Chronic Inflammation on Thermogenic Genes in BAT and WAT in Mice

**Figure 3:**
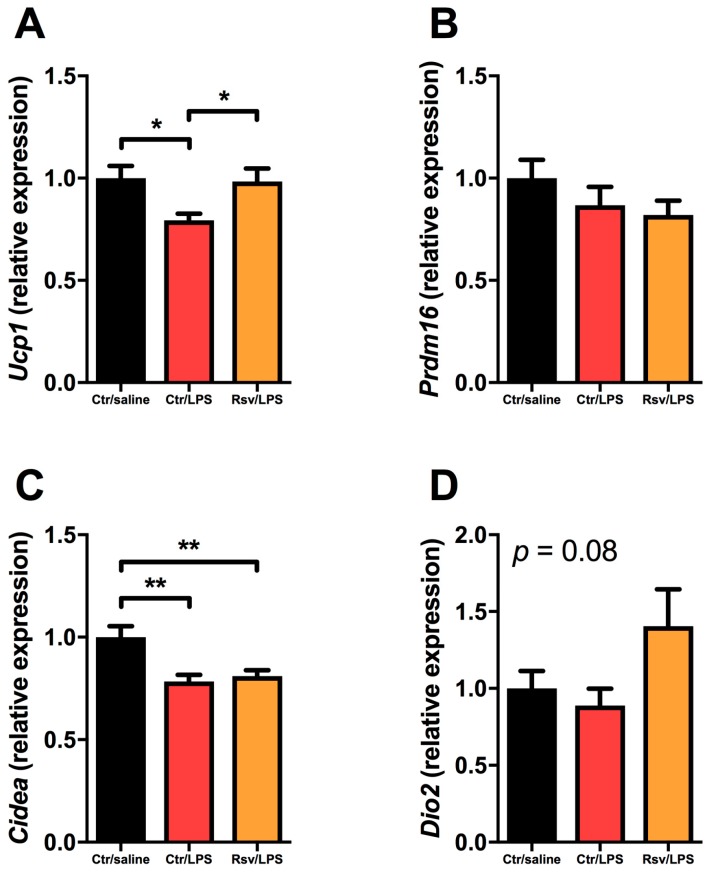
The effects of selected brown genes’ expression in BAT in mice treated with LPS via osmotic mini-pumps and resveratrol through the diet for 28 day. (**A**) *Ucp1*; (**B**) *Prdm16*; (**C**) *Cidea*; and (**D**) *Dio2* expression in control, LPS-treated, or LPS- and resveratrol-treated mice (*n* = 14 to 15). * denotes *p* < 0.05, ** denotes *p* < 0.01 according to one-way ANOVA followed by Bonferroni’s post hoc analysis.

To investigate LPS-mediated alterations of brown genes in BAT and subcutaneous WAT (scWAT), we used a murine model where LPS was infused for 28 days via osmotic mini-pumps [28]. Harvested interscapular BAT showed decreased expression of *Ucp1* by LPS treatment, which was reversed by resveratrol (Figure 3A). Furthermore, LPS reduced the expression of *Cidea* (Figure 3C), but not *Prdm16* (Figure 3B) and *Dio2* (Figure 3D). Resveratrol showed no significant effect on *Prdm16*, *Cidea*, or *Dio2* expression (Figure 3B–D).

**Figure 4:**
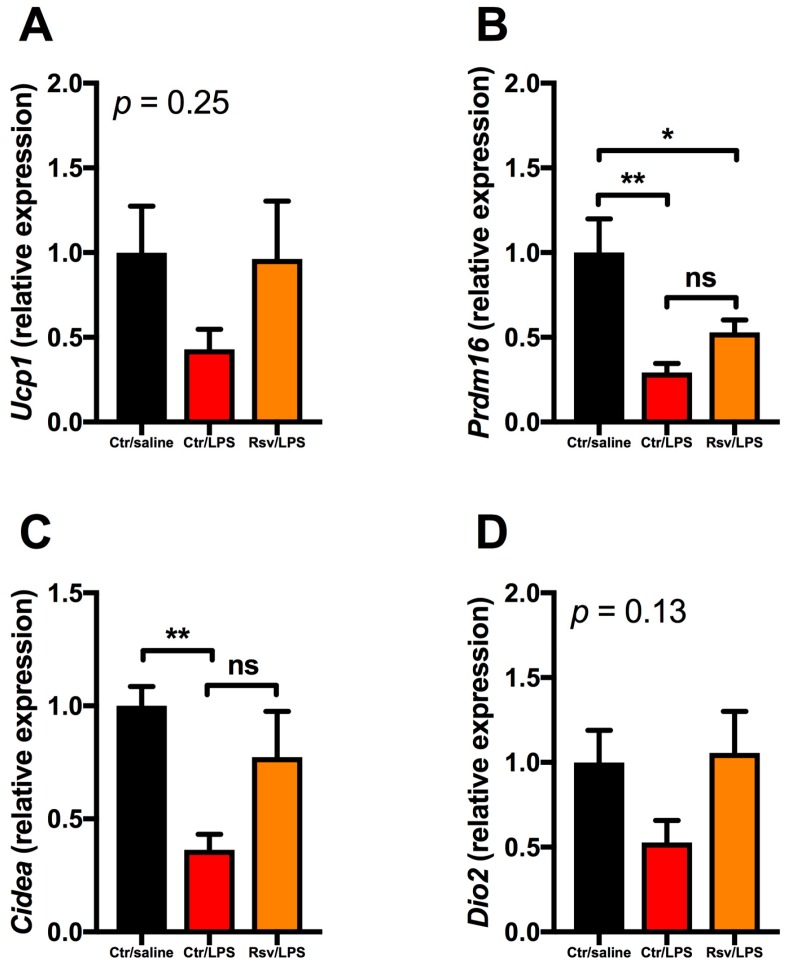
Gene expression of thermogenic genes in subcutaneous white adipose tissue in mice treated with LPS via osmotic mini-pumps and resveratrol through the diet for 28 day. (**A**) *Ucp1*; (**B**) *Prdm16*; (**C**) *Cidea*; and (**D**) *Dio2* expression in control, LPS-treated, or LPS- and resveratrol-treated mice (*n* = 10). * denotes *p* < 0.05, ** denotes *p* < 0.01 according to one-way ANOVA followed by Bonferroni’s post hoc analysis.

In scWAT from mice treated with LPS, there was a similar inhibitory pattern on the thermogenic genes (Figure 4). The decrease in *Ucp1* (Figure 4A) and *Dio2* (Figure 4D) expression did not reach statistical significance, whereas the inhibition of *Prdm16* and *Cidea* after LPS treatment was significant (Figure 4B,C). For all thermogenic genes in WAT resveratrol seemed to attenuate the LPS induced inhibition (albeit not statistically significant) (Figure 4).

### 2.5. Expression of Tlr4 in Brown Adipocytes

**Figure 5:**
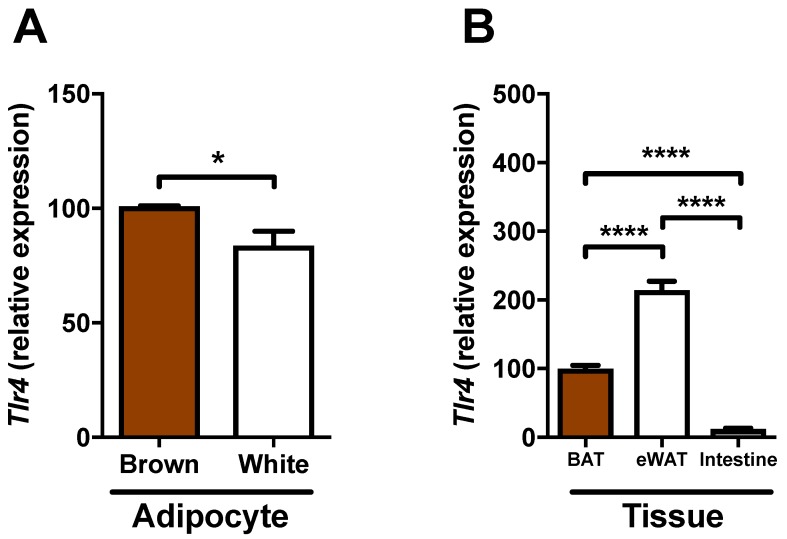
Expression of *Tlr4* in adipocytes and whole tissue. (**A**) Expression of *Tlr4* in mature immortalized brown adipocytes and white 3T3-L1 adipocytes (*n* = 6 to 7); (**B**) Expression of *Tlr4* in BAT, epididymal adipose tissue, and small intestine (ileum) of wild-type C57BL/6 mice (*n* = 10 to 17). * denotes *p* < 0.05, **** denotes *p* < 0.0001 according to one-way ANOVA followed by Bonferroni’s post hoc analysis.

As we found no effects of LPS on brown adipocyte induction in vitro, as opposed to our findings in vivo of *Ucp1*, we questioned whether brown adipocytes express TLR4, which is the receptor mediator of LPS-effects. Previously, white adipocytes have been reported to express TLR4 [6]. Thus, to get an impression of TLR4 gene expression in our brown adipocytes, we combined it with the expression in 3T3-L1 cells originally isolated from white adipose tissue. Brown adipocytes showed a higher *Tlr4* expression compared to white adipocytes (Avg. Ct: 26.6 (brown) vs. 27.1 (white); Figure 5A). Additionally, we compared the *Tlr4* expression of various tissues in normal C57BL/6 mice; epididymal (white) adipose tissue (eWAT) showed the highest expression compared to BAT, which had a higher expression than intestine (Figure 5B).

Despite the expression of *Tlr4*, we could not detect any inflammatory response of LPS treatment on expression of the NF-κB target genes *Il1b* and *Tnfa* in BAT cells. However, LPS stimulation of 3T3-cells elicited an inflammatory response. Similarly, IL1β stimulation induced a robust rise in the inflammatory status of 3T3-cells (TNFα and IL1β mRNA levels), whereas IL1β stimulation in BAT did not increase the mRNA levels of TNFa and IL1β (data not shown).

## 3. Discussion

In this investigation, we showed the reduced expression of *Ucp1* and *Cidea* in mice continuously infused with LPS for 28 days. In immortalized brown adipocytes, incubation with low-dose LPS (2 and 20 ng/mL) did not affect the induction of brown genes. However, incubation with IL1β greatly reduced the induction of *Ucp1* and other brown genes. SIRT1 is probably an important mediator of *Ucp1* induction as *Dbc1* (SIRT1 inhibitor) was upregulated by IL1β incubation and co-incubation with resveratrol (SIRT1 activator) reduced the detrimental effects of IL1β on *Ucp1* induction.

We found a reduced expression of brown genes in mice chronically infused with low-dose LPS (Figure 3) and, in agreement with a recent publication [10], we report the gene expression of the LPS sensing receptor TLR4 by brown adipocytes (Figure 5). Thus, brown adipocytes are probably capable of sensing LPS, but we did not find a direct effect of LPS in mature BAT cells in vitro. In contrast, we found that IL1β reduces the expression of brown genes in mature BAT cells in vitro. This opens the possibility that the observed reduced *Ucp1* expression in vivo by LPS is not directly caused by LPS stimulation of adipocytes, but mediated indirectly via LPS stimulation of macrophages within the tissue to release catecholamines [29], a hypothesis that was recently challenged as macrophages seem to lack the rate-limiting enzyme tyrosine hydroxylase [30], or by LPS stimulating the inflammatory tone and, thus, eliciting other cell types, either locally or more distant to release IL1 that subsequently inhibits UCP1 expression. In support of the latter, recent reports have suggested an important role of macrophage-derived IL1β in diminished browning of white adipocytes in an extracellular signal-regulated kinase-dependent manner [31,32]. Our study supports this notion as we found that chronic inflammation by LPS treatment of the mice generally resulted in a decreased expression of the thermogenic genes in subcutaneous WAT and *Prdm16* (a marker of browning), indicating that LPS treatment diminish browning of WAT.

Surprisingly, treatment of BAT cells in vitro with either IL1β or LPS did not result in transcription of NF-κB target genes (*Il1b*, *Tnfa*) suggesting brown adipocytes are immunologically naïve and do not participate in the escalation of inflammatory responses. In white adipocytes, LPS and IL1β treatment results in increased expression of proinflammatory markers such as monocyte chemoattractant protein-1, interleukin-6, and TNFα [33,34]. However, despite the lack of inflammatory response in brown adipocytes, IL1β does modulate expression of *Ucp1*.

**Figure 6:**
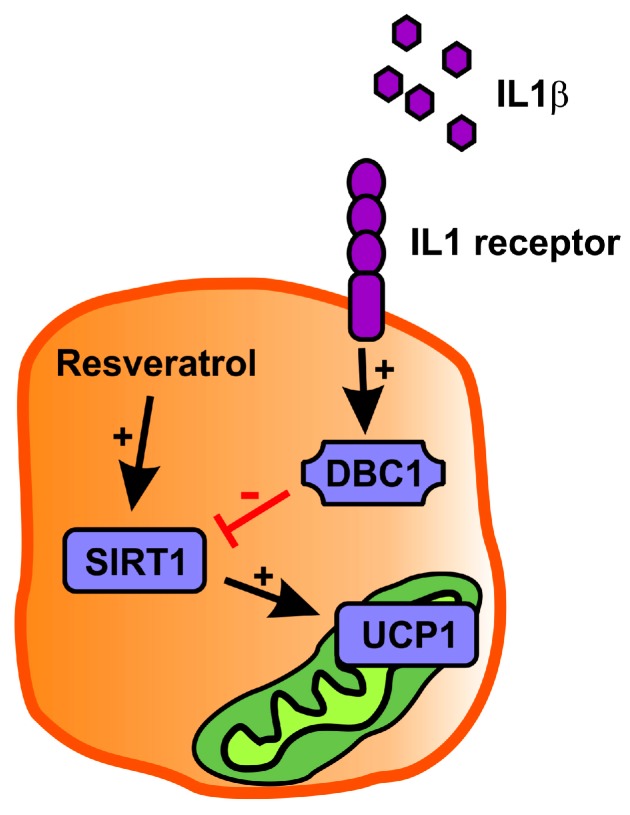
The suggested hypothesis of IL1β-mediated regulation of UCP1 in brown adipocytes. IL1β stimulation upregulates the expression of the SIRT1 inhibitor DBC1. SIRT1 has, here, and previously [17], been shown to be an important regulator of UCP1. Furthermore, enhancing the SIRT1 activity by, e.g., resveratrol, can upregulate the expression of UCP1. Arrow: stimulation; T bar: inhibition.

An interesting observation was the expression pattern of the endogenous SIRT1 inhibitor DBC1. Stimulation (with dbcAMP) of brown adipocytes resulted in downregulation of *Dbc1*, which was partly reversed by IL1β. Previously, the expression of *Ucp1* has been described to be under influence of SIRT1 activity [17], which we also confirmed in mature brown adipocytes (Figure 1C). It is, therefore, intriguing to suggest that IL1β might control the expression of UCP1 via altering the expression of DBC1 (Figure 6).

In support of SIRT1 as a regulator of UCP1 expression, we saw that resveratrol, a known SIRT1 activator, partly rescued the *Ucp1* gene expression following LPS and IL1β treatment in either in vivo or in vitro experiments, respectively. Resveratrol has previously been described to increase energy expenditure in mice [35], which could be explained by the increased expression of *Ucp1*, as we report. Regulation of SIRT1 by resveratrol is a direct effect resulting in increased acetylase activity of the SIRT1 enzyme [23], whereas SIRT1 expression is not commonly regulated by resveratrol [27]. This is in accordance with our findings as we did not detect any effect of LPS or resveratrol on SIRT1 mRNA expression in vivo.

Here, we have shown that low-grade inflammation could be a potential mechanism behind reduced UCP1 expression, which could ultimately lead to obesity or worsen the consequences of obesity. In addition, we have provided data showing that the *Ucp1* expression is regulated by the SIRT1 activity, as DBC1 (SIRT1 inhibitor) and resveratrol (SIRT1 activator) showed opposing effects on *Ucp1* expression. We suggest the manipulation of the SIRT1 activity and its potentially-coupled regulation of UCP1 could be a possible future drug target in anti-obesity treatment.

## 4. Materials and Methods

### 4.1. Cell Cultures

Murine immortalized brown preadipocytes [26] (a kind gift from Bruce Spiegelman, Boston, MA, USA) were grown to approximately 80% confluence in growth medium consisting of Dulbecco Modified Eagle Medium (DMEM) supplemented with 20% fetal calf serum (FCS) and 1% pen/strep. The medium was changed every second day. For initiation of the differentiation into mature brown adipocytes, cells were changed to a differentiation medium (day 0) consisting of DMEM, 10% FCS, 1% pen/strep, 0.02 μM insulin, 5 μM dexamethasone, 125 μM indomethacin, 1 nM T3, and 500 μM isobutylmethylxanthine (IBMX) for 48 h after which the cells were changed (day 2) to the differentiation medium omitting dexamethasone, indomethacin, and IBMX. After five days of differentiation, the cells were ready for experiment procedure.

The incubation period with LPS (*Escherichia coli* serotype 0111:B4, Sigma, St. Louis, MO, USA), IL1β (Sigma, St. Louis, MO, USA) and resveratrol (Evolva, Copenhagen, Denmark) was 24 h at 37 °C. After 20 h of treatment, and cells were supplemented with 500 μM dbcAMP for 4 h to induce the thermogenic program. Concentrations of the used compounds were: LPS: 2 and 20 ng/mL, IL1β: 2 ng/mL, and resveratrol: 12.5 and 25 μM. Resveratrol was diluted in dimethyl sulfoxide.

3T3-L1 cells were grown and differentiated as previously published [33].

### 4.2. Gene Silencing

Silencing of SIRT1 gene expression was obtained with Lipofectamine 2000 Reagent (Invitrogen, Carlsbad, CA, USA) according to manufacturer’s instructions. *Sirt1* (MSS234959) or control (4390843) siRNA (ThermoFisher Scientific, Waltham, MA, USA) was added to the cells at day 2 of the differentiation process and incubated for 20 h. At day 3, the medium was changed and the cells were incubated for further 24 h (day 4) and subjected to the treatment protocol. Cells were harvested at day 5.

### 4.3. Animal Experiments

A murine model of low-grade inflammation was used to test the effect of inflammation and resveratrol on BAT. The procedures have previously been published [28]. Briefly, C57BL/6N mice were subcutaneously implanted with osmotic mini-pumps which infused either LPS (*Escherichia coli* serotype 055:B55, Sigma) at a dose of 600 μg/kg/day or vehicle (saline) for 28 days. Mice had ad libitum access to water and control (Ctr) or resveratrol (Rsv) diet (24% protein, 12% fat, 64% carbohydrates) throughout this treatment period. The Rsv diet consisted of 4 g Rsv per kg chow diet. Interscapular BAT, eWAT, scWAT, and intestine (ileum) were harvested and frozen for gene expression analysis. Animal experiments were approved by the Danish Council for Animal Experiments (No.: 2013-15-2934-00899) and followed the guidelines given in the European Communities Directive of 24 November 1986 (86/609/ECC).

### 4.4. Gene Expression

For extraction of total RNA from cell cultures, cells were briefly incubated in Trizol reagent (Invitrogen), collected in tubes, and frozen for later RNA isolation. For tissue RNA extraction, tissues were homogenized in Trizol reagent with a mixer mill. RNA isolation, reverse transcription, and quantitative PCR (qPCR) followed the principles published previously [28]. Primer sequences for qPCR were designed with QuantPrime [36] and can be found in Table S1. Generally, *Rplp0* was used as a housekeeping gene except for Figure 3 (*Polr2a*) and Figure 4 and Figure 5A (*Gapdh*).

### 4.5. Statistics

Data are presented as means ± standard error of the mean (SEM). Differences of means were evaluated by Student’s *t*-test or ANOVA, followed by a post hoc test where appropriate. Calculation of *p* values and the generation of figures were performed with GraphPad Prism v. 7.0B (GraphPad Software Inc., La Jolla, CA, USA).

## Supplementary Materials

Supplementary materials can be found at www.mdpi.com/1422-0067/18/5/1006/s1.

## Abbreviations

BAT	Brown adipose tissue
cAMP	Cyclic adenosine monophosphate
CIDEA	Cell death activator CIDE-A
DBC1	Deleted in breast cancer 1
dbcAMP	Dibutyryl cyclic adenosine monophosphate
DIO2	Type 2 iodothyronine deiodinase
IL1β	Interleukin 1 β
LPS	Lipopolysaccharide
NF-κB	Nuclear factor κ B
PGC1α	PPARγ co-activator 1 α
PPARγ	Peroxisome proliferator-activated receptor γ
PRDM16	PR domain containing 16
SIRT1	Sirtuin-1
TLR4	Toll-like receptor 4
TNFα	Tumor necrosis factor α
UCP1	Uncoupling protein 1
WAT	White adipose tissue
