# Supplementary material for: Inflammation Downregulates UCP1 Expression in Brown Adipocytes Potentially via SIRT1 and DBC1 Interaction
Source: Int J Mol Sci. 2017 May 8;18(5):1006. doi: 10.3390/ijms18051006 (PMC5454919; doi:10.3390/ijms18051006)
Supplement: Supplementary file 1 [file ijms-18-01006-s001.pdf]

Table S1. Primer pairs used in this investigation.

Gene	Primer	Secuence (5' -> 3')
Cidea	Forward	CCTGCAGGAACTTATCAGCAAGAC
	Reverse	CGTAACCAGGCCAGTTGTGATG
Dio2	Forward	ACAGGTTAAACTGGGTGAAGATGC
	Reverse	GCTCTGCACTGGCAAAGTCAAG
Gapdh	Forward	TTGATGGCAACAATCTCCAC
	Reverse	CGTCCCGTAGACAAAATGGT
Il1b	Forward	CCTGTGTAATGAAAGACGGCACAC
	Reverse	ATTGCTTGGGATCCACACTCTCC
Pgc1a	Forward	CCGTAAATCTGCGGGATGATGGAG
	Reverse	TCAAGAGCAGCGAAAGCGTCAC
Polr2a	Forward	TCCTGGTGAAGACAATGAAGG
	Reverse	TCATAGACATGCGTAAGCCG
Prdm16	Forward	CACGGTGAAGCCATTCATATGCG
	Reverse	AGGTTGGAGAACTGCGTGTAGG
Rplp0	Forward	CTGGAAGTCCAACTACTTCCT
	Reverse	CATCATGGTGTTCTTGCCCAT
Sirt1	Forward	CGATGACAGAACGTCACACGC
	Reverse	TCGAGGATCGGTGCCAATCA
Tlr4	Forward	GCTTGAATCCCTGCATAGAGGTAG
	Reverse	AGGAATGTCATCAGGGACTTTGC
Tnf	Forward	TTCCCAAATGGCCTCCCTCTCATC
	Reverse	TCCTCCACTTGGTGGTTTGCTAC
Ucp1	Forward	GCCATCTGCATGGGATCAAACC
	Reverse	TCGTCCCTTTCCAAAGTGTTGAC

Primer sequences
